# The impact of stress, recovery and coping on burnout symptoms of young elite table-tennis players: A prospective multilevel study

**DOI:** 10.3389/fpsyg.2022.1007697

**Published:** 2022-12-08

**Authors:** Guillaume Martinent, Valérian Cece, Emma Guillet-Descas

**Affiliations:** ^1^Laboratory of Vulnerabilities and Innovation in Sport (EA 7428), University of Claude Bernard Lyon 1 – University of Lyon, Lyon, France; ^2^University of Teaching Education, State of Vaud (HEP Vaud), Lausanne, Switzerland

**Keywords:** athlete burnout, coping, multilevel analyses, recovery, stress

## Abstract

**Objective:**

The aim of the present study was to explore the role of stress, recovery, and coping on table-tennis athlete burnout symptoms in considering both the roles of individual and contextual (training center) factors.

**Methods:**

One hundred and fifty-nine youth elite table-tennis players (*Mage* = 14.07, *SD* = 2.13) involved in 15 intensive training centers completed self-report questionnaires and socio-demographic data.

**Results:**

When time 1 (T1) levels 1 (individual) and 2 (training group, contextual factor) stress, recovery, and coping were simultaneously entered as predictors of each of the three burnout symptoms (physical and emotional exhaustion, sport devaluation, reduced accomplishment) at T2 (controlling for levels 1 and 2 burnout at T1), the results of multilevel analyses revealed that: (a) T1 level 1 recovery significantly negatively predicted T2 reduced accomplishment (*β* = −0.23, *p* = 0.03); (b) T1 level 2 disengagement-oriented coping significantly negatively predicted T2 reduced accomplishment (*β* = −0.71, *p* = 0.03); and (c) T1 level 2 task-oriented coping marginally significantly positively predicted T2 physical and emotional exhaustion (*β* = 0.99, *p* = 0.06).

**Conclusion:**

Results of the present study provided evidence for the usefulness to disentangle the variances attributable to the individual (level 1) and contextual (level 2; training group) levels of the predictors (recovery, stress and coping) of athlete burnout. Moreover, rather than examining the antecedent role of stress on athlete burnout, it could be particularly fruitful to explore theoretical constructs able to annihilate the maladaptive effects of chronic stress such as coping and recovery.

## Introduction

Young elite athletes in intensive training settings must commit to a significant amount of training to accede to the highest competitive levels. Within intensive training centers, they have to manage a series of physical (e.g., injury risks), psychological (e.g., demonstrating personal competence), and social (e.g., distance from family) daily stressors in a win-at-all-coast atmosphere (Martinent et al., 2020). In particular, table-tennis players are confronted to specific psychological constraints related to the limited margin of error, the important number of required repetitions, and the pressure inherent to the competitive environment (Martinent et al., 2014a). These daily stressors may lead to athlete burnout (Martinent et al., 2014b) which can be defined as a syndrome characterized by reduced sense of accomplishment, sport devaluation, and emotional and physical exhaustion (Raedeke, 1997). Burnout is related to maladaptive outcomes such as illness or dropout (Gustafsson et al., 2011). Although prolonged experience of stress can be conceptualized as an antecedent of athlete burnout (Gustafsson et al., 2011), it is noteworthy that a certain level of stress is an integral part of elite sport settings (Martinent and Decret, 2015a). As such, the objective is no longer to annihilate stress but to attempt to reach a balance between stress state and personal resources (e.g., recovery, coping).

The concept of recovery has received increasing attention in research and practice over the past 20 years based on the rationale that it helps understanding how to cope with stress and how to build enduring resources (Kellmann, 2010). Biopsychological perspective of recovery and stress (Kellmann et al., 2018) “embraces physical and biopsychosocial dimensions of both stress and recovery to indicate the extent to which an athlete is physically and/or mentally stressed, as well as whether this athlete is capable of using individual strategies for recovery and which strategies are used” (Nicolas et al., 2022, pp. 1). For instance, recovery allows athletes supporting high loads of daily training and improving their overall fitness whereas the absence of recovery can lead to overtraining and burnout (Kellmann, 2010). Thus, simultaneously measuring stress and recovery states seems particularly salient to assess individual biopsychosocial balance able to foster high-level performance and prevent the triggering of athlete burnout (Kellmann, 2010; Vacher et al., 2017; Kellmann et al., 2018).

Burnout may also depend on athletes’ ability to cope with their daily stressors (Martinent et al., 2014b). Coping can be defined as the set of cognitive strategies and behavioral efforts carried out by athletes to handle the internal and/or external sports requirements that threat to surpass their perceived resources (Nicholls and Polman, 2007). Sport scholars conceptualized coping construct using three core coping dimensions (Gaudreau and Blondin, 2002). Task-oriented coping (TOC) involves strategies that directly face the stressful situation, and the thoughts and affects that appear in the situation (thought control, seeking support, relaxation, logical analysis). Distraction-oriented coping (DsOC) comprises the strategies that focus on other stimuli instead of the stressful one to disconnect from the stressful situation (distancing, mental distraction). Disengagement-oriented coping (DgOC) refers to the strategies allowing to escape from the stressful situation (resignation, venting of unpleasant emotions; Martinent and Nicolas, 2016). Young athletes use fewer coping strategies and are less flexible in their coping range than older athletes (Nicholls and Polman, 2007). Thus, young athletes in intensive training settings could be particularly vulnerable to the stressors encountered in their daily life and in turn to athlete burnout (Martinent and Decret, 2015a). As such, it could be particularly useful to explore the respective impact of stress, recovery and coping strategies on athlete burnout symptoms in including simultaneously these three constructs within the design of the study. Past studies have generally reported positive associations of the use of TOC with adaptive outcomes such as positive affect or sport performance and the use of DgOC with maladaptive outcomes such as negative affect (Gaudreau and Blondin, 2002; Nicholls and Polman, 2007; Doron and Martinent, 2021). Moreover, previous longitudinal studies have highlighted the critical role that coping plays in the development of athlete burnout (Madigan et al., 2020; Pires and Ugrinowitsch, 2021). Results of this literature showed that the use of DgOC was linked to an increase in athlete burnout over time, while TOC was unrelated or negatively associated with changes in burnout over time.

The sport literature also suggested that athlete burnout may result from both personal (stress, recovery, and coping) and contextual factors (Gustafsson et al., 2011). Of particular importance in the context of the present study, the environment in which the young athletes are grounded (intensive training centers) could be conceptualized as a contextual factor likely to impact athlete burnout. Indeed, the atmosphere in the training group could impact recovery, stress and coping factors. For instance, Tamminen and Gaudreau (2014) pointed out an interesting result regarding the social nature of coping processes when athletes deal with shared challenges and demands within a training group characterized by day-to-day influence of teammate interactions on stressors and coping. As such, disentangling the variances attributable to the individual and contextual levels could help clearly depicting the respective roles of individual and contextual factors in the prediction of athlete burnout symptoms. This might provide new insights on the athlete burnout literature likely to bring applied implications related to the prevention of athlete burnout. Distinct strategies aiming at preventing athlete burnout could be implemented if individual or contextual factors predict athlete burnout symptoms.

In sum, the aim of the present study was to explore the role of stress, recovery, and coping on athlete burnout symptoms in considering both the roles of individual and contextual (training center) factors. We hypothesized that: (1) the scores of stress and DgOC would be positively associated with burnout; and (2) the scores of TOC and recovery would be negatively related to burnout symptoms. Moreover, we broadly assumed that the two levels (individual and contextual) would be involved in the prediction of burnout scores.

## Materials and methods

### Participants

One hundred and fifty-nine youth table-tennis players (50 girls, 109 boys; *Mage* = 14.07, *SD* = 2.13; range = 11–19) involved in 15 intensive training centers (from 8 to 22 athletes per centers) accredited by the French Federation of table-tennis participated in this study. These training centers “focus on helping athletes to reach the highest levels of performance, providing the necessary preparation for a successful transition to professional sporting life, and having good academic results” (Martinent et al., 2018, pp. 2726). Participants trained 15.04 h a week (*SD* = 5.78; from 1 to 2 training sessions per day) and their playing experience was 6.36 years (*SD* = 2.24). They participated in regional (*n* = 32), national (*n* = 82), or international (*n* = 45) table-tennis competitions. The competitive schedule of young athletes is based on the school time (main competitive events planned for the end of the school calendar). This study is part of a broader research project focused on different purposes. Thus, the sample of the present study was also used by Martinent and Decret (2015a,b) and Martinent et al. (2014a,b, 2018, 2020). None of the results pertaining to the data in this study are presented elsewhere.

### Measures

The Coping Inventory for Competitive Sport (CICS: Gaudreau and Blondin, 2002) is a French questionnaire of 39 items assessing 10 coping strategies aggregating in the three core dimensions of TOC (thought control, effort expenditure, seeking support, logical analysis, relaxation, mental imagery), DsOC (mental distraction, distancing), and DgOC (venting of unpleasant emotions, disengagement/resignation). Participants rated the use of each coping strategy to cope daily stressors of the past 3 days on a scale of 1 (does not correspond at all) to 5 (corresponds very strongly). Cronbach’s alphas were of 0.86, 0.76 and 0.82 for TOC, DsOC and DgOC, respectively.

The French version of the Recovery-Stress Questionnaire for Athletes (RESTQ-Sport: Martinent et al., 2014b) contains 67 items measuring 17 subscales organized in the two macro dimensions of stress (general stress, emotional stress, social stress, conflicts/pressure, fatigue, lack of energy, physical complaints, disturbed breaks, emotional exhaustion, injury) and recovery (physical recovery, general well-being, sleep quality, being in shape, personal accomplishment, self-efficacy, self-regulation; Kellmann, 2010). Participants rated frequency of each item during past 3 days on a scale ranging from 0 (never) to 6 (always). Cronbach’s alphas were of 0.94 and 0.90 for stress and recovery, respectively.

The French version of the Athlete Burnout Questionnaire (ABQ: Isoard-Gautheur et al., 2010) comprised three subscales assessing reduced accomplishment, sport devaluation, and emotional/physical exhaustion. Participants responded on a 5-point Likert scale ranging from 1 (almost never) to 5 (most of the time). Cronbach’s alphas ranged from 0.66 to 0.89 (for the two measurement times).

### Procedure

The research was approved by the National table-tennis federation’s ethical committee and followed the principles of the Declaration of Helsinki. Permission to contact participants was obtained from the head coaches of each training center. Prior to data collection, written informed consent was gathered from players and their parents. Firstly, participants completed the ABQ, CICS and RESTQ-Sport on the half of the competitive season (Time 1, T1) during which “athletes must cope with ever-increasing social, psychological and physiological demands” (Martinent et al., 2014b, pp. 1651). Secondly, participants completed the ABQ 3 months later, at the end of the competitive season (Time 2, T2).

### Data analyses

A multilevel analysis approach allowed exploring relationships between study variables. Multilevel models extend multiple regressions to nested (hierarchically structured) data (Vacher et al., 2017). Considering hierarchical structure of the data (Level 1: individuals; Level 2: training groups) allowed unbiased estimates of the parameters (Singer and Willett, 2003). All the analyses were computed using the lme4 package of R. Firstly, the intra-class correlations were examined in computing the null models for the three dimensions of athlete burnout at T2 (i.e., dependent variables of the present study). Secondly, we ran a series of multilevel models in which burnout symptoms at T2 were regressed onto levels 1 and 2 coping and recovery-stress states controlling for levels 1 and 2 burnout symptoms at T1. Group mean centering was used for all Level 1 predictors whereas grand mean centring was used for Level 2 predictors based on the rationale no centering may produce biased point estimates (Doron and Martinent, 2016).

## Results

Descriptive statistics are presented in Table 1. The systematic within-and between-individual variance in the T2 athlete burnout dimensions were computed using the null models (see Table 2). Results indicated that there was substantial level 1 (individual) and level 2 (training group) variance: σ^2^ (i.e., variance in level-1 residual) ranged from 0.49 to 0.87 whereas τ_00_ (i.e., variance in level − 2) ranged from 0.02 to 0.08. Thus, the intra-class correlations (ICC = τ_00_/(τ_00_ + σ^2^)) revealed that level 2 variance represented 4–8% to the total variance whereas level 1 variation accounted for 92–96% to the total variance of the athlete burnout symptoms (Table 2).

**Table 1:** Descriptive statistics of the study variables.

	Mean	Standard deviation
Time 1 Stress	1.82	0.80
Time 1 Recovery	3.64	0.74
Time 1 Task-oriented coping	2.85	0.57
Time 1 Distraction-oriented coping	2.00	0.64
Time 1 Disengagement-oriented coping	2.05	0.72
Time 1 Reduced accomplishment	2.45	0.72
Time 1 Emotional/Physical Exhaustion	1.80	0.89
Time 1 Sport devaluation	3.09	0.88
Time 2 Reduced accomplishment	2.40	0.72
Time 2 Emotional/Physical Exhaustion	2.92	0.98
Time 2 Sport devaluation	1.73	0.78

**Table 2:** Results of the null models.

Model equations	Fixed effects	Random effects		−2*log likelihood
	γ_00_ (ES)	σ^2^ (SD)	τ_00_ (SD)	
Reduced accomplishment = β_0_ + r	2.40*** (0.07)	0.49 (0.70)	0.02 (0.15)	301.2
Physical and emotional exhaustion = β_0_ + r	2.95*** (0.11)	0.87 (0.93)	0.08 (0.28)	384.3
Sport devaluation = β_0_ + r	1.71*** (0.08)	0.57 (0.76)	0.03 (0.18)	323.7
β_0j_ = γ_00_ + U_0J_				

*p < 0.05; **p < 0.01; ***p < 0.001. SE, standard errors; SD, standard deviations; β_0j_ is the average level of burnout symptoms for individuals; γ_00_ = is the group mean of burnout symptoms; σ^2^ = var. (*r*_ij_) variance in level-1 residual (i.e., variance in *r*_ij_); τ_00_ = var. (*U*_0j_) variance in level-2 residual (i.e., variance in *U*_0j_); ***p < 0.001.

Not surprisingly, the largest effects of multilevel models were observed for the effects of T1 levels 1 and 2 burnout on the same burnout variable at T2 (0.42 ≥ *β* ≥ 1.78; Table 3). Of greater interest, when T1 levels 1 and 2 stress, recovery, and coping (TOC, DsOC, and DgOC) were simultaneously entered as predictors of each of the three burnout symptoms (physical and emotional exhaustion, sport devaluation, reduced accomplishment) at T2 (controlling for levels 1 and 2 burnout at T1), the results revealed that: (a) T1 level 1 recovery significantly negatively predicted T2 reduced accomplishment (*β* = −0.23, *p* = 0.03); (b) T1 level 2 DgOC significantly negatively predicted T2 reduced accomplishment (*β* = −0.71, *p* = 0.03); and (c) T1 level 2 TOC marginally significantly positively predicted T2 physical and emotional exhaustion (*β* = 0.99, *p* = 0.06; Table 3).

**Table 3:** Results of the multilevel models.

Model equations	Fixed effects													Random effects		−2*log likelihood
	γ_00_ (SE)	γ_01_ (SE)	γ_02_ (SE)	γ_03_ (SE)	γ_04_ (SE)	γ_05_ (SE)	γ_06_ (SE)	γ_07_ (SE)	γ_08_ (SE)	γ_09_ (SE)	γ_10_ (SE)	γ_11_ (SE)	γ_12_ (SE)	σ^2^ (SD)	τ_00_ (SD)	
T2ReducedAccomplishment = β_0_ + β_1_StressL1 + β_2_StressL2_+_β_3_RecoveryL2 + β_4_RecoveryL1 + β_5_TOCL2 + β_6_TOCL1 + β_7_DsOCL2 + β_8_DsOCL1 + β_9_DgOCL2 + β_10_DgOCL1 + β_11_ T1ReducedAccomplishmentL2 + β_12_T1ReducedAccomplishmentL1 + r	2.42*** (0.05)	0.19 (0.23)	0.04 (0.01)	0.71 (0.67)	−0.23* (0.11)	−0.36 (0.35)	−0.00 (0.12)	−0.10 (0.04)	0.07 (0.01)	−0.72* (0.33)	0.00 (0.11)	1.78*** (0.50)	0.42*** (0.01)	0.30 (0.54)	0.00 (0.00)	223.2
T2SportDevaluation = β_0j_ + β1StressL1 + β_2j_ StressL2_+_β_3_RecoveryL2 + β_4_RecoveryL1+ β_5_TOCL2 + β_6_TOCL1 + β_7_DsOCL2 + β_8_DsOCL1 + β_9_DgOCL2 + β_10_DgOCL1 + β_11_T1SportDevaluationL2+ β_12_T1SportDevaluationL1 + r	1.77*** (0.05)	0.23 (0.25)	0.15 (0.10)	0.40 (0.53)	−0.10 (0.11)	−0.86 (0.55)	0.17 (0.14)	0.02 (0.37)	−0.07 (0.11)	0.04 (0.30)	0.02 (0.11)	0.50^¥^ (0.27)	0.45*** (0.08)	0.35 (0.59)	0.00 (0.00)	248.0
T2Emotional/PhysicalExhaustion = β_0j_ + β_1_StressL1 + β_2_StressL2_+_β3RecoveryL2 + β_4_RecoveryL1 + β_5_TOCL2 + β_6_TOCL1+ β_7_DsOCL2 + β_8_DsOCL1 + β_9_DgOCL2 + β_10_DgOCL1 + β_11_T1Emotional/PhysicalExhaustionL2 + β_12_T1Emotional/PhysicalExhaustionL1 + r	2.92*** (0.07)	−0.36 (0.36)	0.03 (0.14)	−0.86 (0.68)	−0.02 (0.15)	0.99^¥^ (0.53)	0.14 (0.17)	−0.14 (0.42)	−0.09 (0.14)	0.12 (0.36)	0.10 (0.14)	0.82*** (0.28)	0.56*** (0.09)	0.58 (0.76)	0.00 (0.00)	317.1
β_0j_ = γ_00_ + U_0J_; β_1j_ = γ_10_ + U_1J_; β_2j_ = γ_20_ + U_2J_; β_3j_ = γ_30_ + U_3J_; β_4j_ = γ_40_ + U_4J_; β_5j_ = γ_50_ + U_5J_; β_6j_ = γ_60_ + U_6J_; β_7j_ = γ_70_ + U_7J_; β_8j_ = γ_80_ + U_8J_; β_9j_ = γ_90_ + U_9J_; β_10j_ = γ_100_ + U_10J_; β_11j_ = γ_110_ + U_11J_; β_12j_ = γ_120_ + U_12J_																

^¥^*p* < 0.06; *p < 0.05; **p < 0.01; ***p < 0.001. SE, standard errors; SD, standard deviations; TOC, task-oriented coping; DsOC, distraction-oriented coping; DgOC, disengagement-oriented coping; γ_00_ = intercept of level-2 regression predicting; β_0j_; γ_10_, γ_20_, γ_30_, γ_40_, γ_50_, γ_60_, γ_70_, γ_80_, γ_90_, γ_100_, γ_110_, γ_120_ = intercept of level-2 regression predicting β_1j_, β_2j_, β_3j_, β_4j_, β_5j_, β_6j_, β_7j_, β_8j_, β_9j_, β_10j_, β_11j_, β_12j_. ^¥^*p* < 0.10; *p < 0.05; **p < 0.01; ***p < 0.001.

## Discussion

The fact that stress did not significantly predicted athlete burnout suggested that rather than annihilating the stress inherent to competitive sport, it seems more useful to help athletes recovering and/or coping with stress to reach an individual biopsychosocial balance able to prevent the fostering of burnout symptoms. In this perspective, it seems particularly useful to examine the role of theoretical constructs able to buffer the maladaptive effects of chronic (prolonged) stress. Indeed, as hypothesized and confirming previous literature (Kellmann et al., 2018), T1 level 1 recovery significantly and negatively predicted T2 reduced accomplishment. Results also provided evidence of the role of coping in the prediction of burnout symptoms. In particular, T1 level 2 DgOC significantly negatively predicted T2 reduced accomplishment whereas T1 level 2 TOC marginally significantly positively predicted T2 physical and emotional exhaustion. These two results are in contrast with cross-sectional (Nicholls and Polman, 2007; Doron and Martinent, 2017) and longitudinal (Madigan et al., 2020; Pires and Ugrinowitsch, 2021) literature showing that DgOC is generally related to dysfunctional athletes’ outcomes whereas TOC is associated with functional athletes’ outcomes. As such, future research should test again the prospective relationships between coping and athlete burnout to see whether the present results emerge in other samples, or whether there were results specific to the current sample.

Of particular importance in the context of the present study, results of the multilevel analyses provided evidence for the usefulness to disentangle the variances attributable to the individual (level 1) and contextual (level 2; training group) levels of the predictors (recovery, stress and coping) of athlete burnout. From an applied perspective, these results might help psychologists and consultants to prevent detrimental psychological outcomes related to burnout. Based on the rationale that only level 1 recovery (but not level 2) significantly predicted burnout symptoms, coaches of intensive training centers of young elite table tennis players had to prioritize individual recovery strategies to ensure an effective biopsychosocial adjustment leading to athletes’ performance, health and well-being (Nicolas et al., 2022). In contrast, results of the present study showed that only level 2 coping (but not level 1) significantly predicted burnout symptoms. As such, sport psychologists should mainly work on collective or shared coping strategies used by table tennis players within the training group in order to optimize the coping process of youth athletes involved in intensive training centers.

Given the specificity of our sample, future research is needed to replicate the present findings with athletes from different ages, sports, levels or other achievement fields (work). Otherwise, only few significant relationships were observed between stress, recovery, coping and athlete burnout. Nevertheless, it is noteworthy that T1 level 1 and 2 athlete burnout were controlled in the prediction of T2 athlete burnout. Another explanation refers to the timing of data gathering of athlete burnout (3 months between the two times). Because athlete burnout is considered to be an enduring phenomenon, substantial time could be needed to note changes (Martinent et al., 2014b). Thus, the present study could be replicated across the entire competitive season. Common method bias might have distorted the findings as all the study variables were measured using a single source of data (self-report questionnaires). Future research should complement self-report questionnaires with objective (e.g., performance data) or physiological indicators of overtraining (e.g., heart rate variability).

In conclusion, our investigation has shed light new insights on the athlete burnout literature in providing evidence for the usefulness to disentangle the variances attributable to the individual (level 1) and contextual (level 2; training group) levels of the predictors (recovery, stress and coping) of athlete burnout. Moreover, results of the present study highlighted that rather than examining the antecedent role of stress on athlete burnout, it could be particularly fruitful to explore theoretical constructs able to annihilate the maladaptive effects of chronic (prolonged) stress such as coping and recovery. Finally, the present study provided further evidence of the usefulness to examine the social nature of coping processes when athletes (individual or team sports) deal with shared challenges and demands within a training group.

## Data availability statement

The raw data supporting the conclusions of this article will be made available by the authors, without undue reservation.

## Ethics statement

The studies involving human participants were reviewed and approved by French table-tennis federation’s ethical committee. Written informed consent to participate in this study was provided by the participants' legal guardian/next of kin.

## Author contributions

GM: conceptualization, methodology, investigation, and writing—original draft preparation. VC: formal analysis. GM, VC, EG-D: writing—review and editing. All authors have read and agreed to the published version of the manuscript.

## Conflict of interest

The authors declare that the research was conducted in the absence of any commercial or financial relationships that could be construed as a potential conflict of interest.

## Publisher’s note

All claims expressed in this article are solely those of the authors and do not necessarily represent those of their affiliated organizations, or those of the publisher, the editors and the reviewers. Any product that may be evaluated in this article, or claim that may be made by its manufacturer, is not guaranteed or endorsed by the publisher.
